# Modeling intracranial aneurysm stability and growth: an integrative mechanobiological framework for clinical cases

**DOI:** 10.1007/s10237-020-01351-2

**Published:** 2020-06-12

**Authors:** Frederico S. Teixeira, Esra Neufeld, Niels Kuster, Paul N. Watton

**Affiliations:** 1 IT’IS Foundation & ETH Zürich, Zürich, Switzerland; 2 Department of Computer Science, Insigneo Institute for in silico Medicine, University of Sheffield, Sheffield, UK; 3 Department of Mechanical Engineering and Materials Science, University of Pittsburgh, Pittsburgh, USA

**Keywords:** Fluid–solid-growth, Growth, Remodelling, Intracranial aneurysm

## Abstract

We present a novel patient-specific fluid-solid-growth framework to model the mechanobiological state of clinically detected intracranial aneurysms (IAs) and their evolution. The artery and IA sac are modeled as thick-walled, non-linear elastic fiber-reinforced composites. We represent the undulation distribution of collagen fibers: the adventitia of the healthy artery is modeled as a protective sheath whereas the aneurysm sac is modeled to bear load within physiological range of pressures. Initially, we assume the detected IA is stable and then consider two flow-related mechanisms to drive enlargement: (1) low wall shear stress; (2) dysfunctional endothelium which is associated with regions of high oscillatory flow. Localized collagen degradation and remodelling gives rise to formation of secondary blebs on the aneurysm dome. Restabilization of blebs is achieved by remodelling of the homeostatic collagen fiber stretch distribution. This integrative mechanobiological modelling workflow provides a step towards a personalized risk-assessment and treatment of clinically detected IAs.

## Introduction

Intracranial aneurysm (IA), a localized focal out-pouching of the cerebral vasculature, occurs in 3–5% of the adult population. The risk of rupture is very low; however, if rupture does occur, mortality rates are very high. Physicians select IAs for intervention by weighing up rupture likelihood with risks associated with treatments. Unfortunately, personalized rupture risk assessment remains elusive and, consequently, intervention may be recommended for IAs that are stable.

Despite continuous efforts on identifying the etiology of the disease, the exact causes of aneurysm formation, growth, stabilization and/or rupture are still not fully understood. Correlations with genetic factors [such as tissue disorders, polycistic kidney disease, first-degree family history etc. (Samuel and Radovanovic 2019)], gender (Desai et al. 2019), ethnicity (Detmer et al. 2019) and external factors (such as high blood pressure, smoking, drugs abuse, head trauma or brain tumor, infection of the arterial wall) have been reported (Toth and Cerejo 2018). Here, we focus on modelling the flow-mediated mechanisms (Cebral et al. 2016) that influence IA pathobiology.

Recent developments in imaging technology have led to asymptomatic IAs being detected more frequently (Ventikos et al. 2009). Hence there is an increasing need to identify IAs that are stable and not in need of intervention. This will reduce the (increasing) financial burden of the disease and will help to reduce unnecessary intervention. Computational models of IAs that quantify their mechanobiological state (and it’s potential evolution) may provide insight into IA stability and thus assist personalized diagnosis.

Computational mechano-biological models of IAs need to account for: (1) the biomechanics of the arterial/aneurysm wall; (2) the cellular biology of the tissue and (3) the dynamic interplay between (1) and (2), i.e. the mechanobiology. Over a decade ago, (Humphrey and Taylor 2008) emphasized the need for a new class of *Fluid-Solid-Growth* models to study aneurysm evolution and proposed the terminology FSG models. These combine fluid and solid mechanics to quantify the flow and structural mechanical stimuli that drive mechanobiological processes. FSG models have been applied by several research groups to model abdominal aortic aneurysm (Zeinali-Davarani and Baek 2012; Watton et al. 2012; Aparício et al. 2014; Wu and Shadden 2015; Grytsan et al. 2015) and IA evolution (Watton et al. 2008, 2009a, 2011a, b; Selimovic et al. 2014).

To date, FSG models have explored phenomenological relationships between mechanical stimuli and tissue remodelling that give rise aneurysm evolution. Nevertheless, they provide a framework for integration of cell models and representation of biochemical pathway models of the arterial wall (Harvey et al. 2018; Aparício 2016) which will provide further insight into disease pathophysiology.

Previous FSG models have IA evolution from an initial *healthy* artery. However, from a clinical perspective characterizing the mechanobiolgical stability (and potential evolution) from the clinical detected geometry is what is important. In this work, we present an integrative FSG modelling framework for the personalized quantification of the mechanobiological state of an IA and it’s potential evolution. This has been realized within *Sim4Life* (Neufeld et al. 2013), a Computational Life Sciences (CLS) simulation platform co-developed by the IT’IS Foundation and ZMT Zurich MedTech AG.

We build on previous studies (e.g. Watton et al. 2011b; Selimovic et al. 2014) to incorporate several new features: integrative pipeline from imaging to simulation; application to patient-specific IA geometries; integration of a novel collagen fiber distribution model into FE code; linking growth and remodelling to pulsatile flow metrics; simulation of secondary blebs; adaption of collagen homeostasis to stabilize IA.

This paper is organized as follows: Section 2 reviews the thick-walled structural model, the computational fluid dynamics solver and the growth and remodeling approach. Simulation results are presented in Sect. 3: first for obtaining the initial homeostatic state in patient-specific geometries; secondly for two different flow-driven remodelling scenarios. Finally, Sect. 5 discusses conclusions and limitations of the model.

## Computational framework and mathematical model for the aneurysm evolution

The computational frawework presented is implemented into *Sim4Life* (Neufeld et al. 2013), a state-of-the-art simulation platform for computational life sciences centered around high-resolution, detailed anatomical phantoms functionalized with dynamic physiology and tissue models; image-based modelling is strongly supported. The platform features a modular graphical user interface (GUI), VTK- and OpenGL-based visualization, a Python scripting interface, and a pipeline framework for advanced analysis. It incorporates:

- A robust vessel segmentation software, capable of dealing with diseased vessel geometries and a multitude of imaging modalities, and supporting efficient interactive corrections where automatic segmentation fails.
- A comprehensive pipeline supporting all steps from image-based model creation to results analysis, including: tissue property assignment; shape characterization; fluid dynamics modeling (with physiologically realistic boundary conditions); solid mechanics; growth and remodeling simulation; analysis, post-processing and visualisation.

**Fig. 1:**
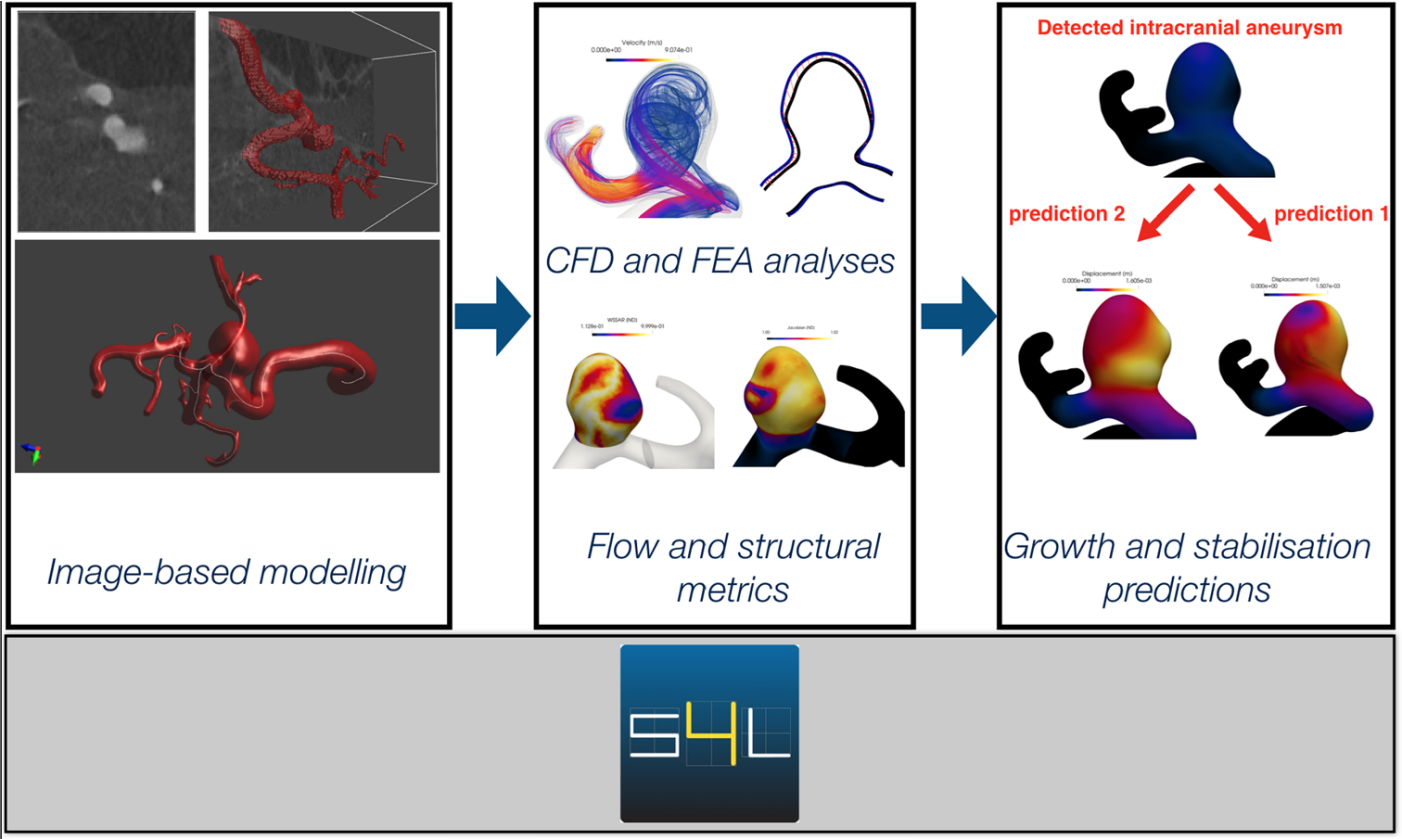
*Sim4Life* integrates all areas of the computational modelling of cerebral aneurysms

It provides the first complete simulation pipeline for modeling growth of clinically detected IA, see Fig. 1.

### Kinematics

The vessel wall experiences large displacement and is made of a nearly incompressible material (Holzapfel 2000). From the structural point of view, it can be decomposed into three layers: intima, media, and adventitia. For a comprehensive analysis of the wall architecture, see Robertson and Watton (2013).

This study models the wall with a the three-dimensional, two-layered thick-wall structure (Teixeira 2019). The endothelial layer is assumed to be mechanically irrelevant, while the medial layer is composed of isotropic materials (elastin, passive smooth muscle ground matrix etc.) and two families of anisotropic collagen fibers; the outer adventitial layer is composed of collagen fibers embedded in an isotropic matrix.

The Lagrangian formulation used in this work denotes the deformation gradient as $\mathbf{F}$, the Green-Lagrange strain tensor as $\mathbf{E}$, and the right Cauchy-Green tensor as $\mathbf{C} = {\mathbf{F}}^T {\mathbf{F}}$. The near-incompressible effects are accounted with a multiplicative split of the deformation gradient $\mathbf{F} = J^{1/3} \bar{\mathbf{F}}$, where $J = \det ( \mathbf{F})$, see Holzapfel et al. (2000). The modified versions of the right Cauchy-Green tensor and Green-Lagrange (GL) strain tensor are defined by $\bar{\mathbf{C}} = \bar{\mathbf{F}}^T \bar{\mathbf{F}}$, and $\bar{\mathbf{E}} = \frac{{1}}{2} \left( \bar{\mathbf{C}} - \mathbf{I}\right)$, respectively. The modified tensor invariants are simply $\bar{I}_1 = tr(\bar{\mathbf{C}})$, $\bar{I}_2 = (tr(\bar{\mathbf{C}})^2- tr(\mathbf{\bar{C}}^2))/2$ and $\bar{I}_3 = det(\bar{\mathbf{C}}) = 1$.

The direction of the collagen fibers is denoted by the unit vector ${{\varvec{m}}}^i$, $i=1,2$. The total stretch $\lambda _4^i$ expresses the total stretch in the direction ${{\varvec{m}}}^i$. The modified version $\bar{\lambda }_4^i = J^{-1/3} \lambda _4^i$ can be evaluated by

1 $$\begin{aligned} \left( \bar{\lambda }_4^i \right) ^2 = {{\varvec{m}}}^i \cdot \bar{\mathbf{C}} {{\varvec{m}}}^i = \bar{I}_4^i \left( \bar{\mathbf{C}}, {{\varvec{m}}}^i \right) , \end{aligned}$$

i.e., associated with $\bar{I}_4^i$, a pseudo-invariant of $\bar{\mathbf{C}}$ and ${{\varvec{m}}}^i$.

We assume the hyperelasticity hypothesis, which implies the existence of an *energy function* $\Psi = \Psi (\mathbf{C})$. The strain energy function $\Psi$ (SEF) is split into an additive sum of isochoric (volume-preserving) and dilatational (volume-changing) parts (Simo et al. 1985)

$$\begin{aligned} \Psi (\bar{I}_1,\bar{I}_2,\bar{I}_4^i, I_3) = \Psi _{iso}(\bar{I}_1,\bar{I}_2) + \Psi _{aniso}(\bar{I}_1,\bar{I}_4^i) + \Psi _{vol}(I_3). \end{aligned}$$

In the absence of external body forces, the displacement field must satisfy $\nabla \cdot \left( \mathbf{F} \mathbf{S} \right) = 0$ where the Second Piola–Kirchhoff stress tensor $\mathbf{S}$ is

$$\begin{aligned} \mathbf{S} = 2 \dfrac{\partial \Psi }{\partial \mathbf{C}} = 2\left( \dfrac{\partial \Psi }{\partial \bar{I}_1} \dfrac{\partial \bar{I}_1}{\partial \mathbf{C}} + \dfrac{\partial \Psi }{\partial \bar{I}_2} \dfrac{\partial \bar{I}_2}{\partial \mathbf{C}} \!+\! \dfrac{\partial \Psi }{\partial \bar{I}_4^i} \dfrac{\partial \bar{I}_4^i}{\partial \mathbf{C}} + \dfrac{\partial \Psi }{\partial {I}_3} \dfrac{\partial {I}_3}{\partial \mathbf{C}} \right) . \end{aligned}$$

### Strain-energy functions

The isochoric part of each layer *L* (where $L=M$ denotes medial layer and $L=A$ denotes the adventitial layer) receives contributions from elastin, passive smooth muscle cells and ground matrix (its isotropic components) and collagen fibers (anisotropic components). The volumetric part receives contributions from the dilatational elastic response of the tissue. Hence

2 $$\begin{aligned} \Psi _L = \Psi _{L,e} + \sum _i \Psi _{L,c}^i + \Psi _{vol}. \end{aligned}$$

#### Elastinous constitutents

The isotropic components are modeled as a neo-Hookean material (Watton et al. 2009b):

3 $$\begin{aligned} \Psi _{L,e} = \left( m_{L,e} \cdot K_{L,e} + m_{L,sm} \cdot K_{L,sm}\right) \cdot \left( \bar{I}_1 - 3\right) , \end{aligned}$$

with $K_{L,e}$ and $K_{L,sm}$ being stiffness-like material constants, and $m_{L,e}$ and $m_{L,sm}$ the (dimensionless) normalized mass density of elastin and passive smooth muscle cells, respectively.

#### Collagenous constituents

The constitutive model for the collagen accounts for the experimental observation that collagen fibers have a a distribution of waviness in the unloaded configuration and thus have a distribution of recruitment (Hill et al. 2012). The strain energy function involves integrating the strain energy for a fiber ($\tilde{\Psi }_{L,c}^i$) over the distribution of recruitment stretches ($\rho ^i_{\rm{rec}}$),

4 $$\begin{aligned} \Psi _{L,c} (\bar{I}_4^i) = m_{L,c} \cdot \sum _i \int _1^{\sqrt{\bar{I}_4^i}} \tilde{\Psi }_{L,c}^i\left( \bar{\lambda }_{4c}^i \right) \rho ^i_{\rm{rec}} \left( \bar{\lambda }_{4r}^i \right) {\rm{d}} \bar{\lambda }_{4r}^i. \end{aligned}$$

where $m_{L,c}$ is the (dimensionless) normalized mass density of collagen fibers that can adapt to simulate growth/atrophy (Watton et al. 2004).

We follow Chen (2014) and model each individual fiber to have a linear relationship between it’s 1st Piola–Kirchoff stress and the collagen fiber stretch $({\bar{\lambda }_{4c}^i})$

$$\begin{aligned} \tilde{\Psi }_{L,c}^i \left( \bar{\lambda }_{4c}^i \right) = \dfrac{K_{L,c}^i}{2} \cdot \left( {\bar{\lambda }_{4c}^i} -1 \right) ^2 \end{aligned}$$

where $K_{L,c}$ is a stiffness-like material constant,

5 $$\begin{aligned} \bar{\lambda }_{4c}^i = {\left\{ \begin{array}{ll} \dfrac{\bar{\lambda }_{4}^i}{\bar{\lambda }_{4r}^i}, &{} \bar{\lambda }_4^i \ge \bar{\lambda }_{4r}^i \\ 1, &{} \text{ otherwise }, \end{array}\right. } \end{aligned}$$

$\bar{\lambda }_{4r}^i = \sqrt{\bar{I}_{4r}^{i}}$ denotes the recruitment stretch of the collagen fiber, and $\bar{\lambda }_4^i =\sqrt{\bar{I}_4^i}$.

We model the fiber recruitment distribution using a triangular probability density function $\rho ^i_{\rm{rec}} \left( \bar{\lambda }_{4r}^i \right)$ (Aparício 2016); $\bar{\lambda }_{4r}^{i,q}$ relates to the the minimum ($q = \min$), modal ($q=mode$) and maximum ($q = {\rm{max}}$) recruitment stretches of the distribution (see Fig. 7). More specifically:

6 $$\begin{aligned} \rho ^i_{\rm{rec}} \left( \bar{\lambda }^i_{4r} \right) = {\left\{ \begin{array}{ll} 0, &{} \bar{\lambda }_{4r}^i \le \bar{\lambda }_{4r}^{i,\min } \\ g_1({\bar{\lambda }_{4r}^i}), &{} \bar{\lambda }_{4r}^{i,\min }< \bar{\lambda }_{4r}^{i} \le \bar{\lambda }_{4r}^{i,{\rm{mode}}} \\ g_2(\bar{\lambda }_{4r}^i), &{} \bar{\lambda }_{4r}^{i,{\rm{mode}}}< \bar{\lambda }_{4r}^{i} \le \bar{\lambda }_{4r}^{i,{\rm{max}}} \\ 0, &{} \bar{\lambda }_{4r}^{i,{\rm{max}}} < \bar{\lambda }_{4r}^i, \end{array}\right. } \end{aligned}$$

where

$$\begin{aligned} g_1(\bar{\lambda }_{4r}^i)= & {} \dfrac{2\left( \bar{\lambda }_{4r}^i - \bar{\lambda }_{4r}^{i,\min }\right) }{\left( \bar{\lambda }_{4r}^{i,{\rm{max}}} - \bar{\lambda }_{4r}^{i,\min }\right) \left( \bar{\lambda }_{4r}^{i,{\rm{mode}}} - \bar{\lambda }_{4r}^{i,\min }\right) } \\ g_2(\bar{\lambda }_{4r}^i)= & {} \dfrac{2 \left( \bar{\lambda }_{4r}^{i,{\rm{max}}} - \bar{\lambda }_{4r}^i \right) }{\left( \bar{\lambda }_{4r}^{i,{\rm{max}}} - \bar{\lambda }_{4r}^{i,\min } \right) \left( \bar{\lambda }_{4r}^{i,{\rm{max}}} - \bar{\lambda }_{4r}^{i,{\rm{mode}}} \right) }. \end{aligned}$$

**Fig. 2:**
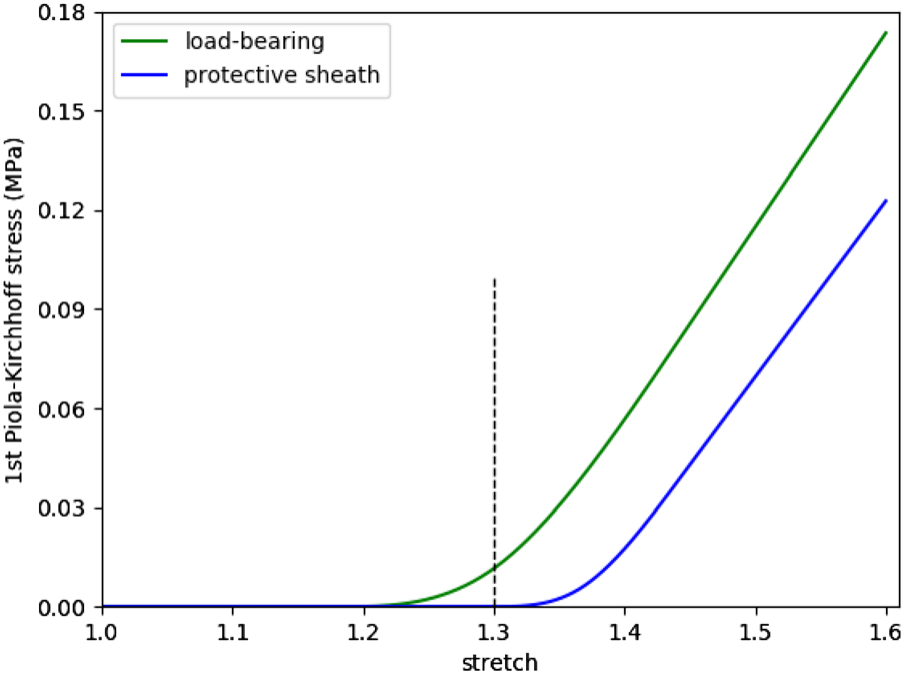
Illustrative example of the collagen fiber stress response. Here medial collagen fibers are recruited to load bearing from a recruitment stretch of 1.18 whilst adventitial collagen fibers begin to be recruited to load bearing at a recruitment stretch of 1.3. Notice the toe region as more of the fibers are recruited to load bearing. Once all fibers are recruited , the relationship between 1st Piola–Kirchoff stress versus stretch becomes linear

Insertion of Eqs. 5 and 6 into  4 and integration yields analytic expressions for the strain energy from which $\dfrac{\partial \Psi }{\partial \bar{I}_4^i}$ can be derived. More specifically:

$$\begin{aligned} \dfrac{\partial \Psi }{\partial \bar{I}_4^i} = \left\{ \begin{array}{lllll} 0, &{} \bar{\lambda }_{4}^i \le \bar{\lambda }_{4r}^{i,\min } \\ h_1(\bar{\lambda }_{4}^{i}), &{} \bar{\lambda }_{4r}^{i,\min }< \bar{\lambda }_{4}^{i} \le \bar{\lambda }_{4r}^{i,{\rm{mode}}} \\ h_2(\bar{\lambda }_{4}^{i}), &{} \bar{\lambda }_{4r}^{i,{\rm{mode}}}< \bar{\lambda }_{4}^{i} \le \bar{\lambda }_{4r}^{i,{\rm{max}}} \\ h_3(\bar{\lambda }_{4}^{i}), &{} \bar{\lambda }_{4r}^{i,{\rm{max}}} < \bar{\lambda }_{4}^i, \end{array} \right. \end{aligned}$$

with

$$\begin{aligned} h_1(\bar{\lambda }_{4}^{i})= & {} \dfrac{1}{\bar{\lambda }_4^i} \left[ c_1 \bar{\lambda }_4^i \log {\left( \bar{\lambda }_4^i \right) } + c_2 \log {\left( \bar{\lambda }_4^i \right) } + c_3 \bar{\lambda }_4^i + c_4 \right] \\ h_2(\bar{\lambda }_{4}^{i})= & {} \dfrac{1}{\bar{\lambda }_4^i} \left[ c_5 \bar{\lambda }_4^i \log {\left( \bar{\lambda }_4^i \right) } + c_6 \log {\left( \bar{\lambda }_4^i \right) } + c_7 \bar{\lambda }_4^i + c_8 \right] , \\ h_3(\bar{\lambda }_{4}^{i})= & {} \dfrac{1}{\bar{\lambda }_4^i} \left[ c_9 \bar{\lambda }_4^i + c_{10} \right] , \end{aligned}$$

where $c_i$ are constants involving $K_{L,c}^i$, $\bar{\lambda }_{4r}^{i,\min }$, $\bar{\lambda }_{4r}^{i,{\rm{max}}}$ , $\bar{\lambda }_{4r}^{i,{\rm{mode}}}$, see appendix of Aparício et al. (2016) for details. Figure 2 illustrates the $1^{st}$ Piola–Kirchhoff stress versus stretch for the fiber-distribution for illustrative recruitment stretch distributions.

#### Volumetric function

The volumetric/dilatational elastic response adopted here stems from Simo and Armero (1992) and is defined as

7 $$\begin{aligned} \Psi _{vol}(I_3) = \dfrac{\kappa }{4} \left[ I_3 - 1 - 2 \log \left( \sqrt{I_3} \right) \right] , \end{aligned}$$

where the parameter $\kappa$ is the bulk modulus.

### Structural solvers

A *PETSc*-based (Balay et al. 2019a, b, 1997) HPC-enabled FEM framework has been created, that flexibly supports a range of element types (tetrahedral, pyramidal, prismatic, etc.), shape-functions, preconditioning schemes, and multi-physics coupling. Meshes are partitioned with *Parmetis* (Karypis 2015). The numerical solution of linear system through Algebraic Multigrid (AMG) Methods relies on HYPRE (Chow et al. 1998).

A specialized structural solver has been developed based on the FEM solver framework. It supports compressible/incompressible, homogeneous/heterogeneous, linear/non-linear, and isotropic/anisotropic material models, multiple populations of collagen fibers with varying orientations and recruitment stretch distributions.

### Computational fluid dynamics

A computational fluid dynamics (CFD) solver was also developed on the basis of the FEM solver framework. It supports $\mathcal{P}_1$-$\mathcal{P}_1$ elements through Variational Multiscale Stabilization (Bazilevs et al. 2007; Forti and Dedè 2015; Liu and Marsden 2018). The algebraic system is solved with FGMRES (Saad 1993), preconditioned with one iteration of SIMPLE (Patankar and Spalding 1983; Pernice and Tocci 2001). Internally, the velocity and Schur complement blocks are preconditioned with fixed iterations of HYPRE (Falgout and Yang 2002).

**Fig. 3:**
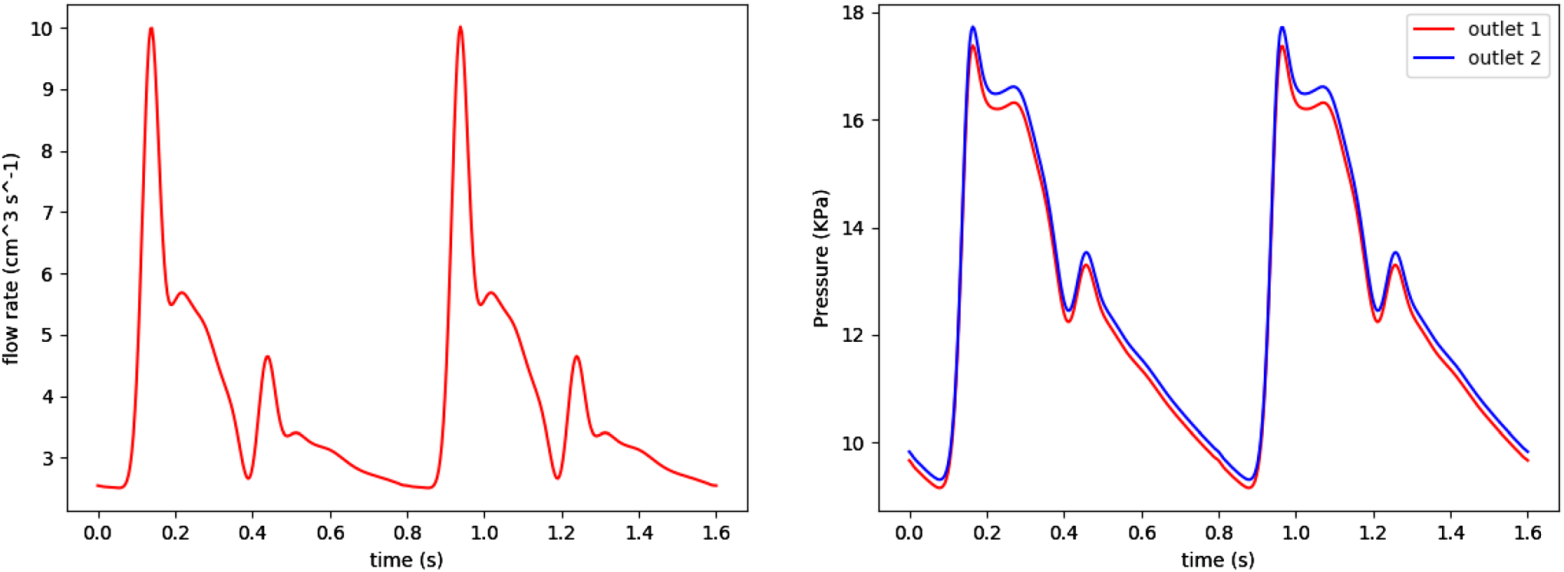
Inlet flow rate (left) and outlet pressure waveforms from (Villa-Uriol et al. 2011) used in the described transient CFD simulations

The incompressible Navier–Stokes equations are used to model flow. The physical parameters of the simulated fluid resemble those of blood: density $\rho _f=$ 1066 kg/m$^3$ and dynamic viscosity $\mu _f=3.5 \times 10^{-3}$ Pa s. Three cardiac cycles of 0.8s each were simulated with time-step 0.004s. Figure 3 plots the inlet flow rate and outlet pressure boundary conditions (Villa-Uriol et al. 2011).

#### Flow metrics

Given the fluid pressure *p* and velocity $\mathbf{v}$, let

$$\begin{aligned} \varvec{\sigma } = -p \mathbf{I} + \mu (\nabla \mathbf{v} + \nabla \mathbf{v}^T) \end{aligned}$$

denote the Newtonian fluid stress tensor, and $\varvec{\eta }$ be an outward unit normal vector at the lumen surface. The wall shear stress vector $\varvec{\tau }$ is given by

$$\begin{aligned} \varvec{\tau } = \varvec{\sigma } \varvec{\eta } - (\varvec{\sigma } \varvec{\eta }) \cdot \varvec{\eta } \end{aligned}$$

and represents the shearing component of the traction vector $\varvec{\sigma } \varvec{\eta }$.

The complex geometry of arteries coupled with pulsatile flow gives rise to regions of the vasculature that are subject to oscillatory flow (magnitude and direction). This can be quantified by a novel flow metric, the wall shear stress aspect ratio (WSSAR) which naturally emerges from quantifying the *shear stress rosettes*, which capture the multi-directionality of flow (Krishna et al. 2020). The WSSAR measures the bidirectionality of the WSS over a given interval [0, *T*] (Krishna et al. 2020). It is calculated as follows: given a point *x* on the wall, let $\varvec{\tau }=\varvec{\tau }(x,t)$ denote the WSS at the point *x* for $t \in [0,T]$, and $\varvec{\eta }_1=\varvec{\eta }_1(x)$ and $\varvec{\eta }_2=\varvec{\eta }_2(x)$ two orthogonal directions which

$$\begin{aligned} \begin{array}{rlrl} \text{maximize} \displaystyle \int _0^T \varvec{\tau }(x,t) \cdot \varvec{\eta }_1(x) {\rm{d}}t &{} \text{ and } \\ \text{minimize} \displaystyle \int _0^T \varvec{\tau }(x,t) \cdot \varvec{\eta }_2(x) {\rm{d}}t. &{} \end{array} \end{aligned}$$

The directions $\varvec{\eta }_1$ and $\varvec{\eta }_2$ are named *principal directions* at point *x*. In addition, let

$$\begin{aligned} \tau _{1,\max }(x)= & {} \max \left\{ \varvec{\tau }(x,t) \cdot \varvec{\eta }_1; ~ \forall t \in [0,T] \right\} \\ \tau _{1,\min }(x)= & {} \min \left\{ \varvec{\tau }(x,t) \cdot \varvec{\eta }_1; ~ \forall t \in [0,T] \right\} \\ \tau _{2,\max }(x)= & {} \max \left\{ \varvec{\tau }(x,t) \cdot \varvec{\eta }_2; ~ \forall t \in [0,T] \right\} \\ \tau _{2,\min }(x)= & {} \min \left\{ \varvec{\tau }(x,t) \cdot \varvec{\eta }_2; ~ \forall t \in [0,T] \right\} \\ \tau _{\max }(x)= & {} \max \left\{ \tau _{2,\max }(x) - \tau _{2,\min }(x), \right. \\&\left. \tau _{1,\max }(x) - \tau _{1,\min }(x) \right\} \\ \tau _{\min }(x)= & {} \min \left\{ \tau _{2,\max }(x) - \tau _{2,\min }(x), \right. \\&\left. \tau _{1,\max }(x) - \tau _{1,\min }(x) \right\} \\ \end{aligned}$$

be auxiliary values, which by definition, satisfy $\tau _{1,{\rm{max}}} \ge \tau _{1,\min }$, $\tau _{2,\max } \ge \tau _{2,\min }$, and $\tau _{\max } \ge \tau _{\min }$. Then the WSSAR, denoted $\tau _{\rm{AR}}$, is defined as

8 $$\begin{aligned} \tau _{\rm{AR}}(x) = \dfrac{\tau _{\min }(x)}{\tau _{\max }(x)}, \end{aligned}$$

and satisfies $0 \le \rm{WSSAR} \le 1$, and

9 $$\begin{aligned} \left\{ \begin{array}{rclrcl} \rm{WSSAR} = 1 &{} \Longleftrightarrow &{} \text{ bi-directional flow } \\ \rm{WSSAR} = 0 &{} \Longleftrightarrow &{} \text{ uni-directional flow. } \end{array} \right. \end{aligned}$$

### Growth and remodelling of the constituents

Clinical studies indicate that the medial layer degrades during aneurysm development, the intima layer disappears, the internal elastic lamina is lost, and apoptosis of vascular smooth muscle cells is observed (Tong et al. 2014). At the same time, the adventitia layer thickens.

For this model, the starting point is an already developed IA. Initial mass-densities of elastin, collagen and smooth muscle cells are prescribed to represent atrophy of the medial layer. Subsequent degradation is driven by linking the mass-densities of constituents to perturations of flow metrics from homeostatic values. Although this approach still does not represent the full complexity of the IA formation with respect to the hemodynamic environment, it is step forward with respect to its application to anatomical IA geometries (Teixeira 2019).

#### Mass degradation

Additional aneurysm enlargement is modeled as a localized degradation of the layer *L* through the decay of the elastin ($m_{L,e}$), collagen ($m_{L,c}$), and smooth muscle cells normalized masses ($m_{L,sm}$) (Watton et al. 2011a). The normalized mass degradation is defined as

10 $$\begin{aligned} \dfrac{\partial m_{L,y}}{\partial t}\left( x,t\right) = - \mathcal{F}_{X}\left( \varvec{\tau }\left( x,t\right) \right) ~ D_{\rm{max}} ~ m_{L,y}\left( x,t\right) , \end{aligned}$$

where $D_{\rm{max}}$ is the maximum rate of degradation, $y=e$ for elastin and $y=c$ for collagen in the layer *L*; $\mathcal{F}_{X}$ represents the influence of hemodynamics on G&R by linking collagen and elastin degradation to WSS-related metrics (Watton et al. 2011b).

The hemodynamic environment of an IA is intimately related to it’s initiation, growth and rupture (Robertson and Watton 2012; Cebral et al. 2019). It is observed that endothelial cells may still be present in developed aneurysms (Froösen et al. 2004 ) and biochemical pathways associated with functional/dysfunctional endothelium are likely to play a role in enlargement and rupture. In the following examples, we consider simple phenomenological relationships between regions of destructive remodelling and perturbed flow; firstly low WSS and, secondly, high oscillatory flow.

**Fig. 4:**
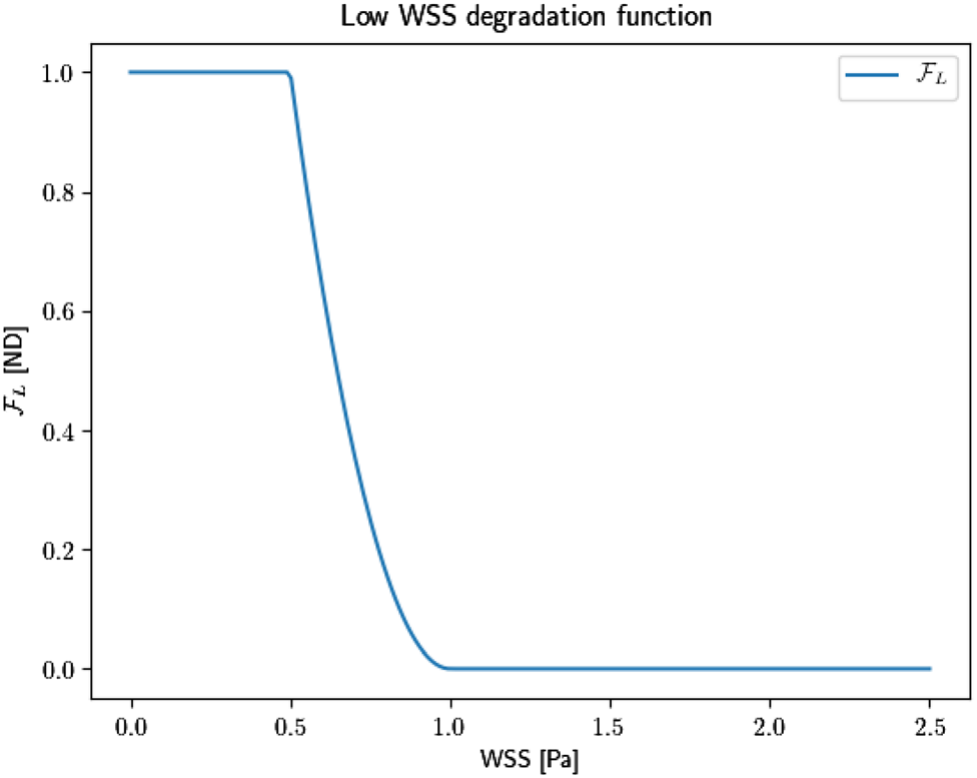
Low WSS degradation function with $\tau _{C} = 1$ Pa and $\tau _L = 0.5$ Pa

*Low WSS*: We follow Watton et al. (2009a), Watton et al. (2011b), Watton et al. (2011a), Selimovic et al. (2014) and assume a simple phenomological relationship between the WSS and degenerative processes of an IA, i.e. without explicitly modelling biochemical pathways. More specifically, $\mathcal{F}_{\rm{WSS}}$ in Eq. 10, is taken to be quadratic:

11 $$\begin{aligned} \mathcal{F}_{\rm{WSS}}\left( \varvec{\tau }\right) = \left\{ \begin{array}{llllll} 1, &{} \vert \vert \varvec{\tau } \vert \vert \le \tau _{L} \\ \left( \dfrac{\tau _{C} - \vert \vert \varvec{\tau } \vert \vert }{\tau _{C} - \tau _L} \right) ^2, &{} \tau _L< \vert \vert \varvec{\tau } \vert \vert < \tau _{C} \\ 0, &{} \tau _{C} \le \vert \vert \varvec{\tau } \vert \vert . \end{array} \right. \end{aligned}$$

where degradation is maximal if $\vert \vert \varvec{\tau } \vert \vert \le \tau _L$, and there is no degradation if $\tau _{C} < \vert \vert \varvec{\tau } \vert \vert$. For illustration, we follow Watton et al. (2009a) and assume that if the WSS is below 1 Pa, it will drive degenerative processes. We set $\tau _{C} = 1$ Pa, $\tau _L = 0.5$ Pa and $D_{\rm{max}} = 1.5$. Figure 4 plots the function $\mathcal{F}_{\rm{WSS}}$ with the above-mentioned values.

*WSSAR*: We hypothesize that endothelial morphology is related to the oscillatory nature of the flow and suppose that there exists a switch point where endothelial cells have distinct phenotypes, i.e. spindle or irregular morphologies. The morphology of the cells may affect the permeability and functionality of the endothelial layer. Specifically, we assume that regions where endothelial cells have irregular morphologies will drive destructive remodelling processes.

The spatially-variable function $\mathcal{F}_{\rm{AR}}\left( \varvec{\tau }\right)$ quantifies the WSSAR (defined as $\tau _{\rm{AR}}$), such that, $\mathcal{F}_{\rm{AR}} = 0$ means no degradation and $\mathcal{F}_{\rm{AR}} = 1$ yields maximum degradation. The existence of thresholds $\tau _{H,{\rm{AR}}}$ is hypothesized, such that the degradation is maximal if $\tau _{H,{\rm{AR}}} \le \tau _{\rm{AR}}$, and absent if $\tau _{\rm{AR}} < \tau _{C,{\rm{AR}}}$. In this case, $\mathcal{F}_{\rm{AR}}$ is given a simple quadratic form as

12 $$\begin{aligned} \mathcal{F}_{\rm{AR}}\left( \varvec{\tau }\right) = \left\{ \begin{array}{llllll} 0, &{} \tau _{\rm{AR}} < \tau _{C,{\rm{AR}}} \\ \left[ \dfrac{\tau _{\rm{AR}} - \tau _{C,{\rm{AR}}}}{\tau _{{\rm{AR}},H} - \tau _{C,{\rm{AR}}}} \right] ^2, &{} \tau _{C,{\rm{AR}}} \le \tau _{\rm{AR}} \le \tau _{H,{\rm{AR}}} \\ 1, &{} \tau _{H,{\rm{AR}}} \le \tau _{\rm{AR}} \end{array} \right. \end{aligned}$$

**Fig. 5:**
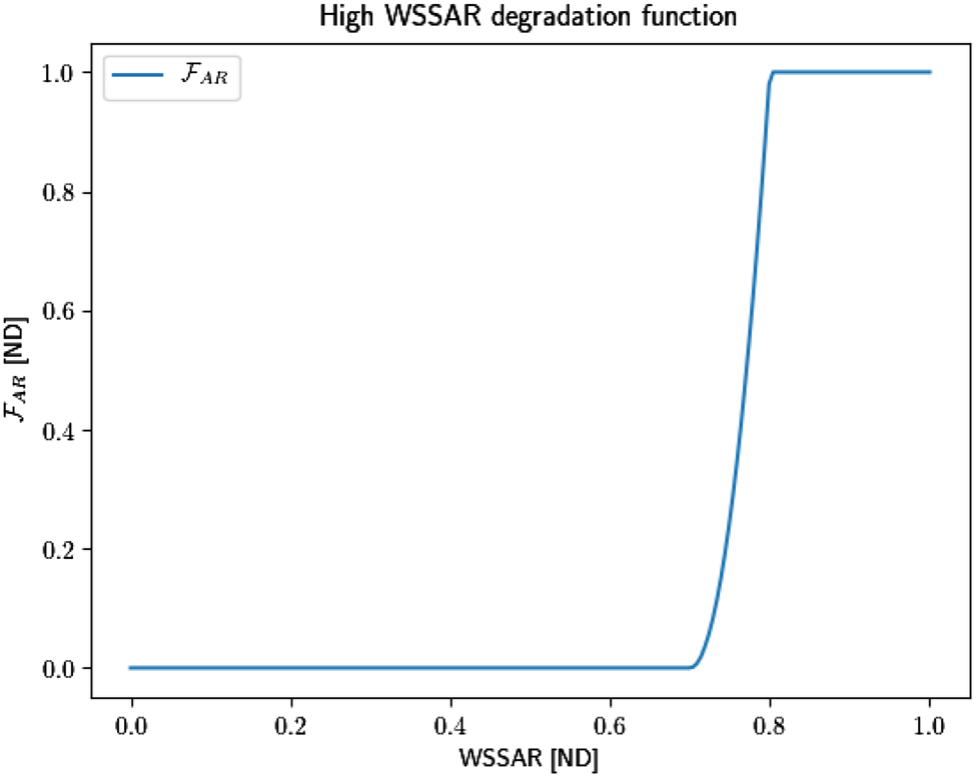
High WSSAR degradation function with $\tau _{C,{\rm{AR}}} = 0.7$ Pa and $\tau _{H,{\rm{AR}}} = 0.8$ Pa

For illustration, we suppose that a WSSAR magnitude of 0.7 acts as a switch point for endothelial cells (ECs) to be packed irregularly. For the simulations, the critical threshold to activate degradation is set to $\tau _{C,{\rm{AR}}} = 0.7$ Pa, while $\tau _{H,{\rm{AR}}} = 0.8$ Pa. $D_{\rm{max}}$ is set to 1.5. Figure 5 plots the function $F_{\rm{AR}}$ with the above-mentioned values.

#### Collagen remodelling

Collagen fibers are in a continuous state of degradation and deposition and are configured to the artery in the physiological configuration in a state of stretch (Humphrey 1999). A consequence of this is that in the unloaded configuration they may appear wavy and thus the tissue needs to be stretched for a wavy fiber to be *recruited* to load bearing. Watton et al. 2004 introduced the terminology: *attachment stretch*, $\bar{\lambda }_{4c,{\rm{att}}}$, to denote the stretch a fiber is configured to the matrix in the loaded configuration; *recruitment stretch*, $\bar{\lambda }_{4r}$, to denote the amount the unloaded tissue must be stretched (in the fiber direction) for the fiber to straighten out and begin to bear load. The collagen fiber stretch is then related to the tissue stretch $\bar{\lambda }^i_4$ by the simple relationship $\bar{\lambda }^i_{4c} = \bar{\lambda }^i_4 / \bar{\lambda }^i_{4r}$—if the tissue is in homeostasis (at physiological stretch) $\bar{\lambda }^i_{4c} = \bar{\lambda }_{{4c,{\rm{att}}}}$.

*In vitro* studies show that there is a distribution of collagen fiber recruitment stretches (Hill et al. 2012). Equivalently, this implies that there exists a distribution of attachment stretches: fibers which are recruited first to load bearing will be configured with maximum attachment stretches and fibers which are last to be recruited to load bearing will have minimum attachment stretches. Chen (2014) generalized the remodelling approach of Watton et al. (2004) to account for remodelling of a distribution of fibers. The initial distribution of recruitment ($t=0$) is determined by assuming a prescribed range of fiber attachment stretches. Remodelling of the recruitment stretch distribution then acts to maintain the collagen fiber stretch distribution (in the loaded configuration) towards the attachment stretch distribution, simulating the natural consequence of fiber deposition/degradation. Mathematically, this is achieved by evolving the recruitment stretch distribution so that the collagen stretch distribution remodels towards the attachment stretch distribution (Aparício et al. 2016):

13 $$\begin{aligned} \begin{aligned} \dfrac{\partial \bar{\lambda }_{4r}^{i,\min }}{\partial t}&= \alpha _0 \left( \dfrac{\bar{\lambda }_{4c}^{i,{\rm{max}}} - \bar{\lambda }_{{4c,{\rm{att}}}}^{i,{\rm{max}}}}{\bar{\lambda }_{{4c,{\rm{att}}}}^{i,{\rm{max}}}} \right) \Big |_{\rm{max}(dias,sys)}\\ \dfrac{\partial \bar{\lambda }_{4r}^{i,{\rm{mode}}}}{\partial t}&= \alpha _0 \left( \dfrac{\bar{\lambda }_{4c}^{i,{\rm{mode}}} - \bar{\lambda }_{{4c,{\rm{att}}}}^{i,{\rm{mode}}}}{\bar{\lambda }_{{4c,{\rm{att}}}}^{i,{\rm{mode}}}} \right) \Big |_{\rm{max}(dias,sys)}\\ \dfrac{\partial \bar{\lambda }_{4r}^{i,{\rm{max}}}}{\partial t}&= \alpha _0 \left( \dfrac{\bar{\lambda }_{4c}^{i,\min } - \bar{\lambda }_{{4c,{\rm{att}}}}^{i,\min }}{\bar{\lambda }_{{4c,{\rm{att}}}}^{i,\min }} \right) \Big |_{\rm{max}(dias,sys)} \end{aligned} \end{aligned}$$

where we assume collagen fibers remodel to achieve a maximum stretch during the cardiac cycle and we assume that this will occur either at systole or diastole (Watton et al. 2011a).

We define three thresholds, namely $\bar{\lambda }_{{4c,{\rm{att}}}}^{i,\min }$, $\bar{\lambda }_{{4c,{\rm{att}}}}^{i,mean}$ and $\bar{\lambda }_{{4c,{\rm{att}}}}^{i,{\rm{max}}}$. The fibers with lowest attachment stretch $\bar{\lambda }_{{4c,{\rm{att}}}}^{i,\min }$ will be recruited last and therefore have the highest recruitment $\bar{\lambda }_{4r}^{i,{\rm{max}}}$, while fibers attached with $\bar{\lambda }_{{4c,{\rm{att}}}}^{i,{\rm{max}}}$ are recruited first and have the smallest recruitment stretch $\bar{\lambda }_{4r}^{i,\min }$. This implies that the collagen fiber recruitment stretches form a distribution, with the thresholds defined as

$$\begin{aligned} \bar{\lambda }_{4c}^{i,{\rm{max}}} = \dfrac{\bar{\lambda }^i_4}{\bar{\lambda }_{4r}^{i,\min }}, \quad \bar{\lambda }_{4c}^{i,{\rm{mode}}} = \dfrac{\bar{\lambda }^i_4}{\bar{\lambda }_{4r}^{i,{\rm{mode}}}}, \quad \bar{\lambda }_{4c}^{i,\min } = \dfrac{\bar{\lambda }^i_4}{\bar{\lambda }_{4r}^{i,{\rm{max}}}}. \end{aligned}$$

#### Collagen growth

The collagen fiber growth is modeled by the evolution of the normalized mass $m_{L,c}$. We assume that (Watton and Ventikos 2009):

- The number of fibroblasts is proportional to the mass of collagen they are maintaining.
- Fibroblasts evolve their stress-free configuration as the collagen fabric remodels and they configure to the matrix to achieve a homeostatic stretch $\bar{\lambda }^i_{4f,\rm{att}}$.
- Collagen mass growth/atrophy is driven by deviation of fibroblast stretch from the homeostatic levels.

The simplest equation to model this phenomenon is

14 $$\begin{aligned} \dfrac{\partial m_{L,f}^i}{\partial t} = \epsilon _L^i m_{L,f}^i \left( \dfrac{\bar{\lambda }_{4f}^{i} - \bar{\lambda }_{4f,\rm{att}}^{i}}{\bar{\lambda }_{4f,\rm{att}}^{i}} \right) \Big |_{sys} \end{aligned}$$

where $\epsilon _L^i>0$ is a growth rate parameter. We assume that the natural configuration of a fibroblast cell to be identical to the natural configuration of the collagen fibers (with maximum stretch) and so $\bar{\lambda }_{4f}^{i} = \bar{\lambda }_{4c}^{i}$ and the above equation can be written in terms of the collagen fiber stretch.

#### Collagen stabilization mechanism

The adventitia acts as a protective sheath against overdistension of an artery due to natural variations in systemic pressure (Schmid et al. 2013). The implication being that it’s load-bearing role at physiological pressures may be negligible (age, species, location dependent). Conversely, the parent dome of an intracranial aneurysm can consist of a thin collagenous sac which bears all the pressure load at physiological pressures, i.e. it is not acting as a protective mechanical sheath. These observations imply that the collagen fiber stretch distribution for the sac will differ from that of the protective sheath. Moreover, the fact that IAs generally stabilize in size implies that the collagen fabric is in mechanobiological equilibrium and thus the adventitial collagen fabric homeostasis adapts—suggesting that fibers are configured to bear more load as the load-bearing role of the medial layer is lost.

We simulate this by remodelling the distribution of attachment stretches (Mandaltsi 2016). Here we do not model how this is biologically achieved—rather we simply prescribe its adaption to achieve a new homeostasis for secondary blebs. The numerical implementation is as follows: we shift the attachment stretch towards current collagen stretch values by prescribing

15 $$\begin{aligned} \bar{\lambda }_{{4c,{\rm{att}}}}^{i,q} \Big |_{t_{n+1}} = \bar{\lambda }_{{4c,{\rm{att}}}}^{i,q} \Big |_{t_{n}} + \alpha _{\rm{att}} \left( \bar{\lambda }_{4c}^{i,q} \Big |_{t_{n+1}} - \bar{\lambda }_{{4c,{\rm{att}}}}^{i,q} \Big |_{t_{n}} \right) \end{aligned}$$

where $q=$ max,mode,min and $\alpha _{\rm{att}}$ is a remodelling rate parameter which controls how quickly the attachment stretch adapts. It is set to 0.5 for the analyses in the following sections.

### Heterogeneous characterization of the arterial wall

**Fig. 6:**
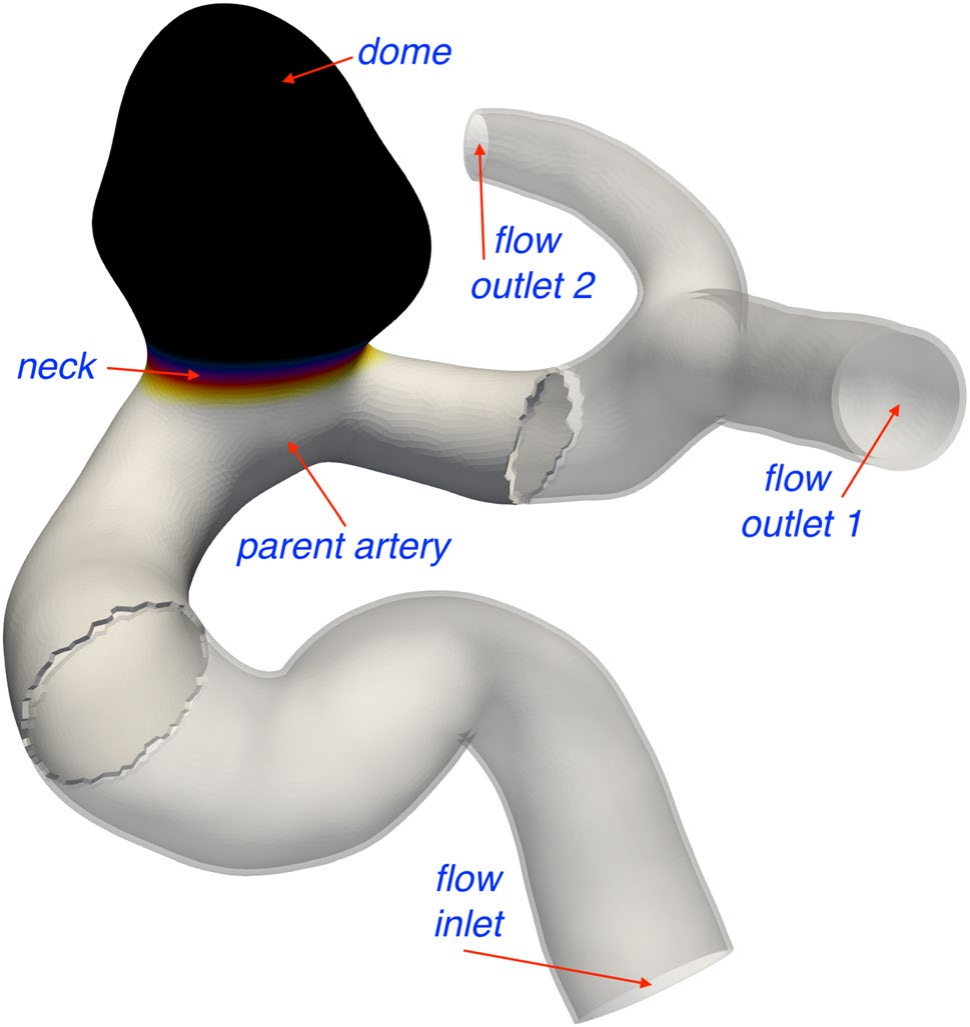
Complete geometry for CFD and the reduced domain (the neighborhood of the dome region) simulated in the structural analysis

The geometry (obtained from Prof. Robertson, University of Pittsburgh) was extracted from computed tomography (CT). The generation of surface and volumetric meshes were performed in *Sim4Life*. In order to reduce the computational burden of the structural analysis, the computation was restricted to the neighborhood of the dome region. The planar cuts in the parent artery were selected far enough from the dome to avoid extra stresses caused by the clamped boundaries. The domain of interest was further split into *parent artery*, *neck* and *dome* subregions in order to locally adapt the mechanical characterization of the wall. Figure 6 shows the whole geometry and depicts each subregion.

#### Physiological parametrization

**Fig. 7:**
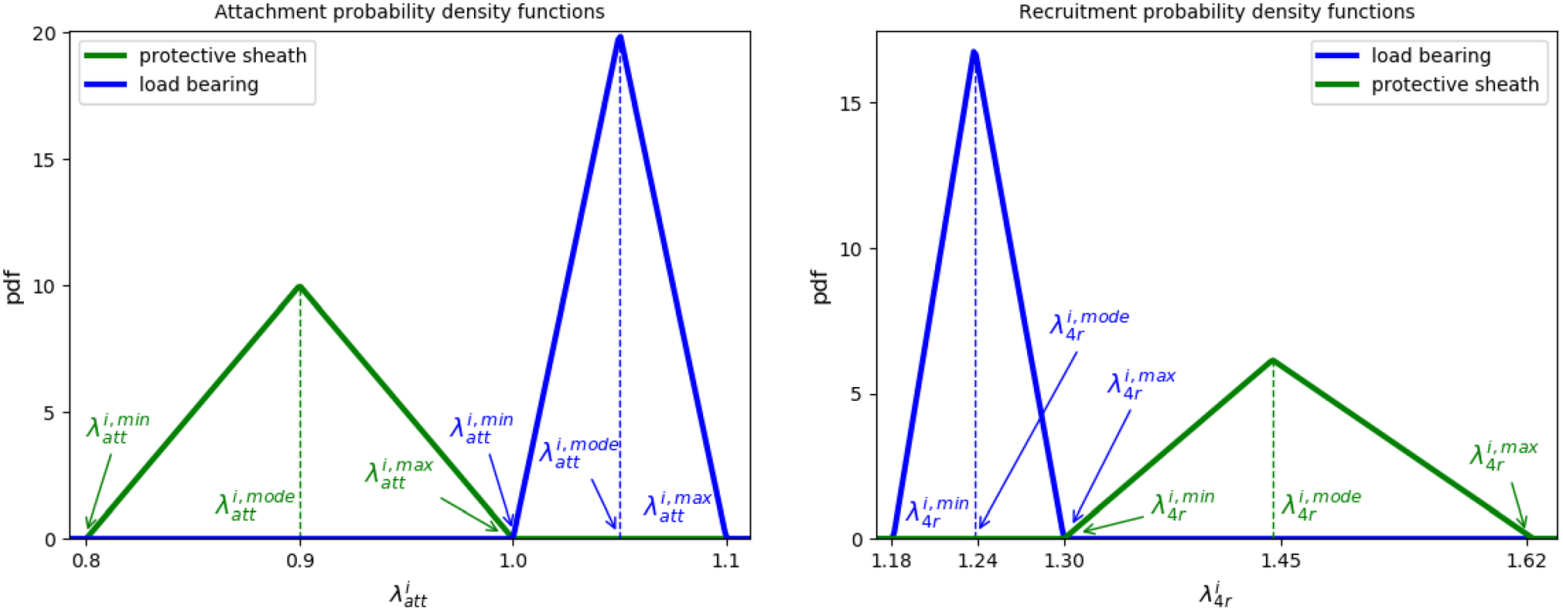
Illustrative probability density functions for load-bearing (blue) and protective (green) adventitial collagen attachment stretches (left), and the corresponding recruitment stretches (right) assuming a circumferential stretch of 1.3

Tables 1 and 2 specify the list of parameters for the mathematical model (see Sect. 2 and Teixeira (2019) for a detailed description). The values reflect the physiological function of each layer for the wall for distinct healthy (*parent artery*) and diseased regions (aneurysm *dome*). For example, in the *parent artery*, the adventitia is assumed to act as a protective sheath (Schmid et al. 2013), and therefore the collagen fibers in this layer are only recruited at supraphysiological pressure. On the other hand, the adventitia acts as the main load-bearing component in the aneurysm *dome* (due the degradation of the medial layer), and therefore the collagen fibers must be recruited within the physiological range of pressures so that they are load-bearing (Robertson and Watton 2013). The initial recruitment stretch values are determined so that collagen achieves a prescribed homeostatic fiber stretch distribution at $t=0$; Fig. 7 illustrates attachment and recruitment stretch distributions using an illustrative stretch of 1.3.

The *neck* region is considered to be a transition region, with parameters being linearly interpolated between the healthy artery and the aneurysm sac.

**Table 1:** List of parameters for the *parent artery* region

Parameter	Value (media)	Value (adventitia)
$K_{L,e}$	0.1 MPa	0.1 MPa
$K_{L,c}^i$	1.0 MPa	1.0 MPa
$\alpha _0$	10.0	10.0
$\epsilon _L^i$	0.0	0.0
$\bar{\lambda }_{\rm{att}}^{i,\min }$	0.95	0.8
$\bar{\lambda }_{\rm{att}}^{i,{\rm{mean}}}$	1.0	0.9
$\bar{\lambda }_{\rm{att}}^{i,{\rm{max}}}$	1.05	1.0
$\bar{\lambda }_{r,\rm{init}}^{i,\min }$	1.01	1.2
$\bar{\lambda }_{r,\rm{init}}^{i,{\rm{mean}}}$	1.05	1.25
$\bar{\lambda }_{r,\rm{init}}^{i,{\rm{max}}}$	1.1	1.3

**Table 2:** List of parameters for the *dome* region

Parameter	Value (media)	Value (adventitia)
$K_{L,e}$	0.1 MPa	0.1 MPa
$K_{L,c}^i$	1.0 MPa	1.0 MPa
$\alpha _0$	10.0	10.0
$\epsilon _L^i$	0.0	0.0
$\bar{\lambda }_{\rm{att}}^{i,\min }$	0.8	1.0
$\bar{\lambda }_{\rm{att}}^{i,{\rm{mean}}}$	0.9	1.05
$\bar{\lambda }_{\rm{att}}^{i,{\rm{max}}}$	1.0	1.1
$\bar{\lambda }_{r,\rm{init}}^{i,\min }$	1.2	1.01
$\bar{\lambda }_{r,\rm{init}}^{i,{\rm{mean}}}$	1.25	1.05
$\bar{\lambda }_{r,\rm{init}}^{i,{\rm{max}}}$	1.3	1.1

#### Collagen fiber orientation

**Fig. 8:**
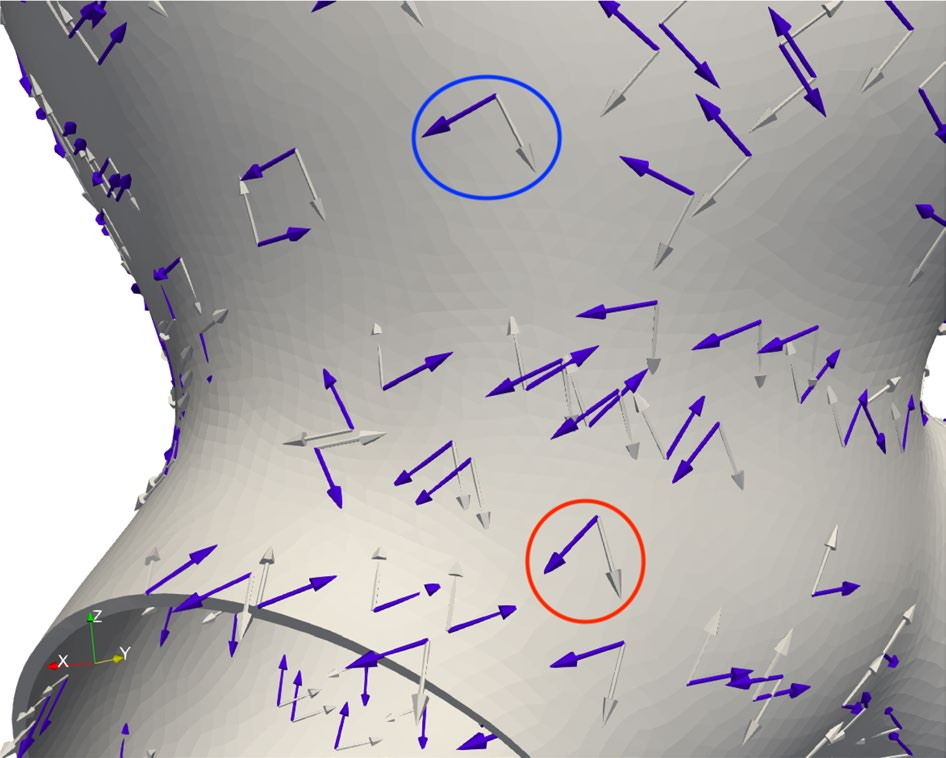
Collagen fiber directions (blue and white arrows) in the *dome* and *parent artery* regions

We model the collagen fiber orientations on the aneurysm *dome* to coincide with the principal curvature directions (Ma et al. 2007), whilst those in the *parent artery* are modeled as splayed about the circumferential direction (Holzapfel et al. 2000). Figure 8 depicts the resulting fiber orientation control: in the *dome* region, the directions were based on the principal curvature; in the *parent artery*, the directions were modified (rotated) $30^\circ$ (in the medial layer) and $60^\circ$ (in the adventitial layer) with respect to the circumferential direction. To maintain continuity of the fiber orientations, we interpolate the fiber directions in the *neck* region.

### Simulation setup

Most IAs stabilize and remain asymptomatic. Hence, when an IA is detected, it is more likely to be stable than unstable. The iterative method to define the *initial homeostasis* in personalized geometries was established in Teixeira (2019). As a next step, the *degradation* phase implements two degradation approaches to model collagen and elastin evolution: (1) linked to low steady-state WSS, and (2) linked to pulsatile flow metrics (see Sect. 2.4.1). Finally, the *stabilization* phase proposes a mechanism based on remodelling the attachment stretches so that the collagen fabric can bear more load in homeostasis without increases in mass. Secondary bleb formation is simulated for a period of 3 years after initial homeostasis: 2 years of degradation linked to flow metrics, and 1 year of stabilization (see Sect. 2.5); The applied time-step of 0.02 year requires a total of 150 steps.

For each time-step of the disease evolution problem, steady-state structural analysis of the reduced domain (see Fig. 6) is performed for diastolic and systolic pressures. The disease evolution model at each point is driven by the maximum $\bar{\lambda }_{4c}^i$, see Eq. (5), of the systolic and diastolic configurations. CFD analyses recompute the WSS field over the whole domain every 20 steps after the onset of degradation (at time-step 50). The steady-state CFD analysis uses a constant inlet flow rate of $2.54 \times 10^{-6} m^3/s$ and constant outlet pressures of 9671.38 Pa and 9833.19 Pa, respectively, which represents the mean flow rate over the cardiac cycle. For the G&R linked to WSSAR, transient CFD analyses runs for 2.4s (three cardiac cycles) with time-step 0.002s (total of 600 time-steps, 200 per cardiac cycle) and uses the physiological inlet flow rate and outlet pressure waves depicted in Fig. 3.

Each full G&R simulation which is linked to steady-state flow took 7 hours on average to run using 10 MPI processes on a Windows desktop computer equipped with an Intel Core *i*9-7900*X* 3.31GHz processor and 64GB of RAM. Those linked to transient flow took 26 hours on average.

## Results

In this section, first we overview the numerical approach to model a stabilized IA. From this homeostatic state, development of secondary blebs is subsequently driven by linking collagen degradation to deviation of flow metrics from homeostatic ranges. Lastly, remodelling of the distribution of collagen fiber attachment stretches stabilizes the blebs.

### Initial homeostasis

Given an initial prescribed recruitment stretch distribution field and established targets for collagen fiber stretch distribution (i.e. the minimum, mode, maximum attachment stretches), an iterative process searches for a (spatially heterogeneous) recruitment distribution field which yields mechanobiological equilibrium for the loaded IA. For the investigated configuration, a simulation with 50 iterations was sufficient to achieve homeostasis.

**Fig. 9:**
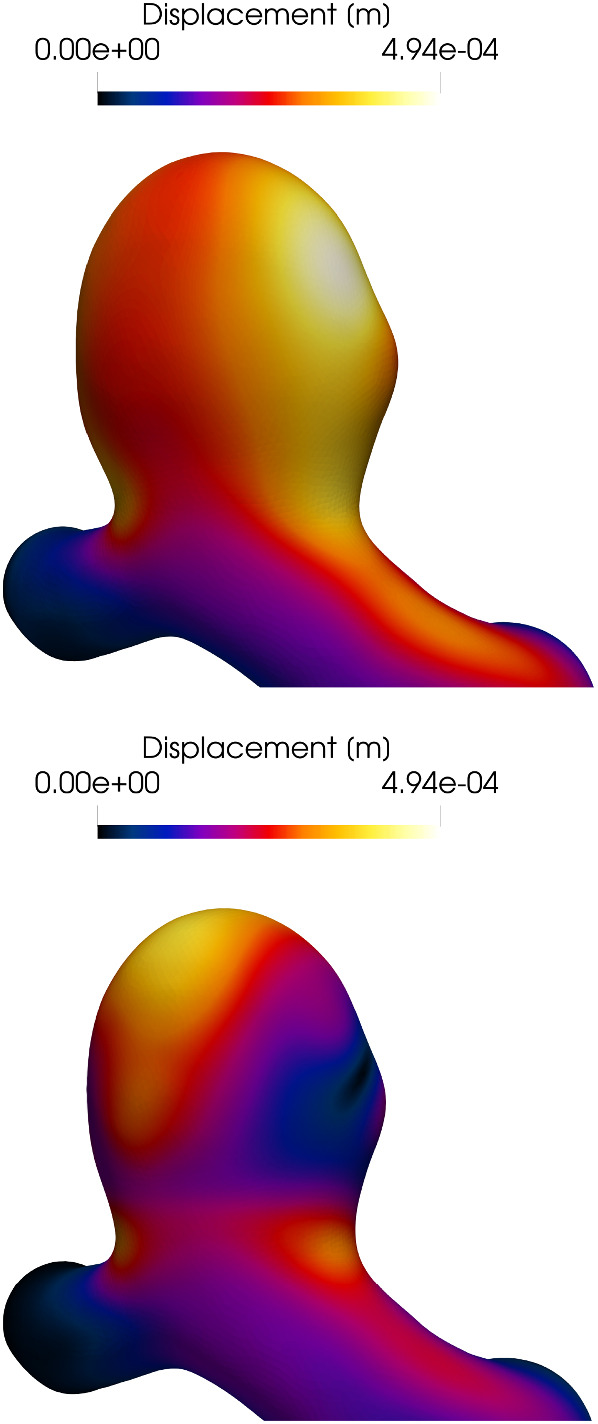
Evolution of the displacement field from the initial loaded geometry (top) to the (initial) homeostatic state (bottom)

**Fig. 10:**
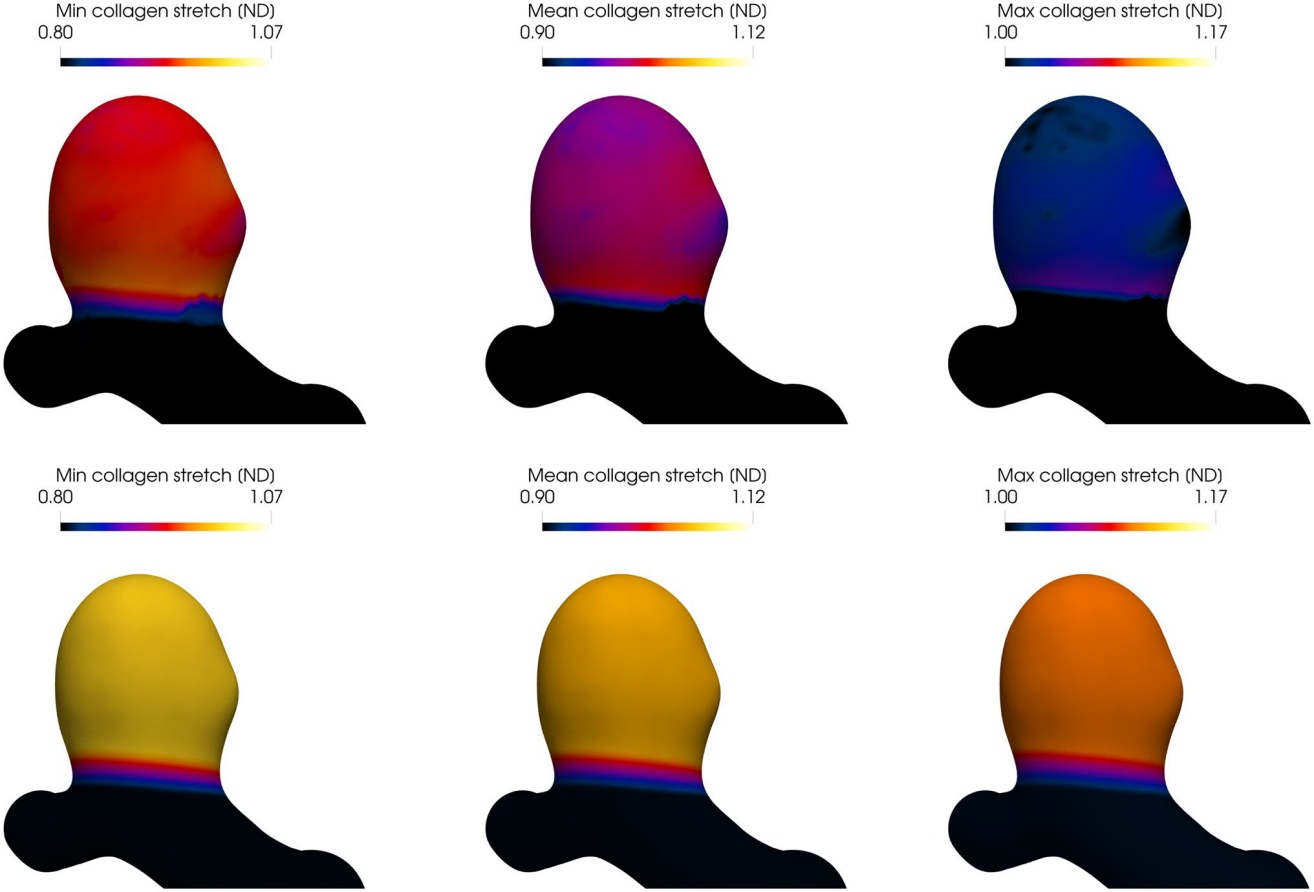
Evolution of the collagen fiber stretch from the initial loaded geometry (first row), to the initial homeostatic state (second row). The first, second and third columns illustrate the minimum, mean and maximum collagen fiber stretches (of the fiber stretch distribution), respectively

Figure 9 depicts the displacement evolution from the *initial deformed geometry* to the *(initial) homeostatic configuration*. Figure 10 depicts the minimum, mode and maximum values of the collagen fiber stretch distribution. Notice that in the homeostatic state, these values are spatially uniform and equal to prescribe homeostatic values (see Table 2) and the collagen fabric is in mechanobiological equilibrium.

**Fig. 11:**
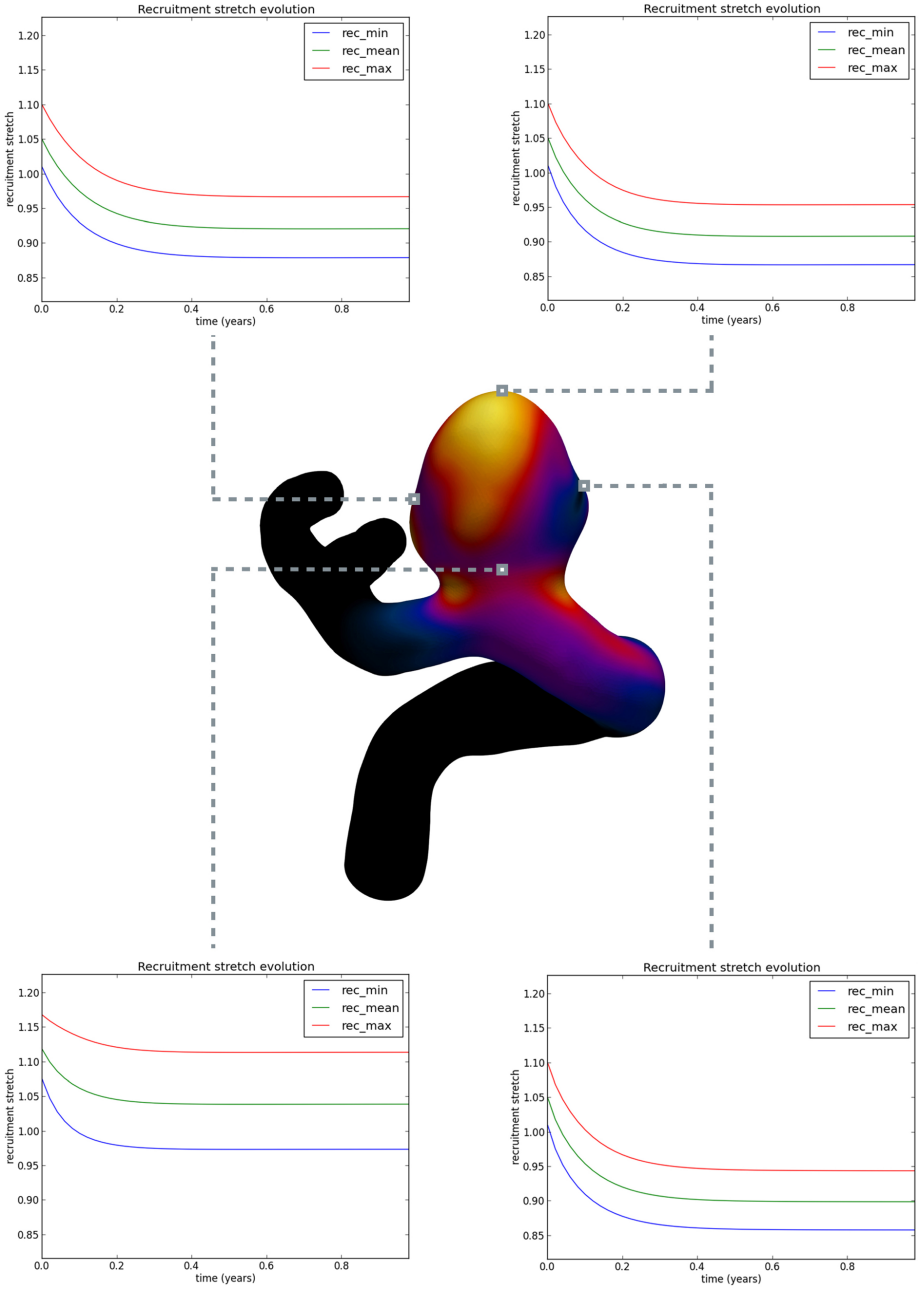
Evolution of the recruitment stretch distribution (from initial prescribed values) to achieve a homeostatic state

**Fig. 12:**
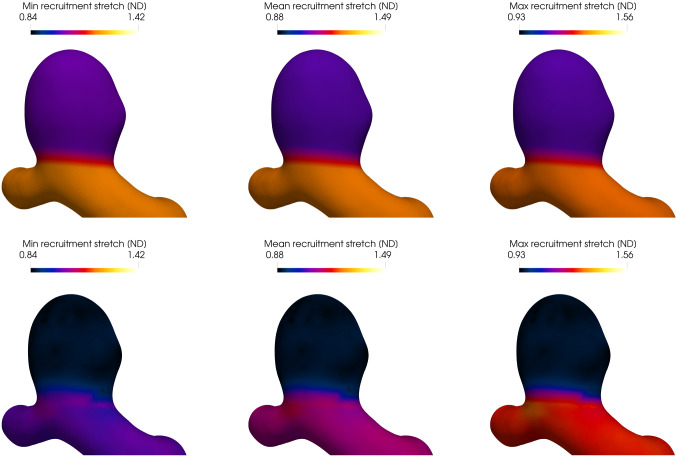
Evolution of the fiber recruitment stretch from the initial deformed geometry (first row), to the initial homeostatic state (second row). The first, second and third columns depict the minimum, mean and maximum collagen recruitment stretches, respectively

The evolution of the recruitment stretch distribution in Fig.  11, driven by Eq. (13), shows that the recruitment stretch distribution was overestimated and decreases so that collagen fiber stretch distribution remodels to the attachment stretch distribution, i.e. via Eq. (6); Fig. 12 illustrates the evolution of the recruitment stretches over the aneurysm sac from the initial pressurized state to the the initial homeostatic state.

### Enlargement

**Fig. 13:**
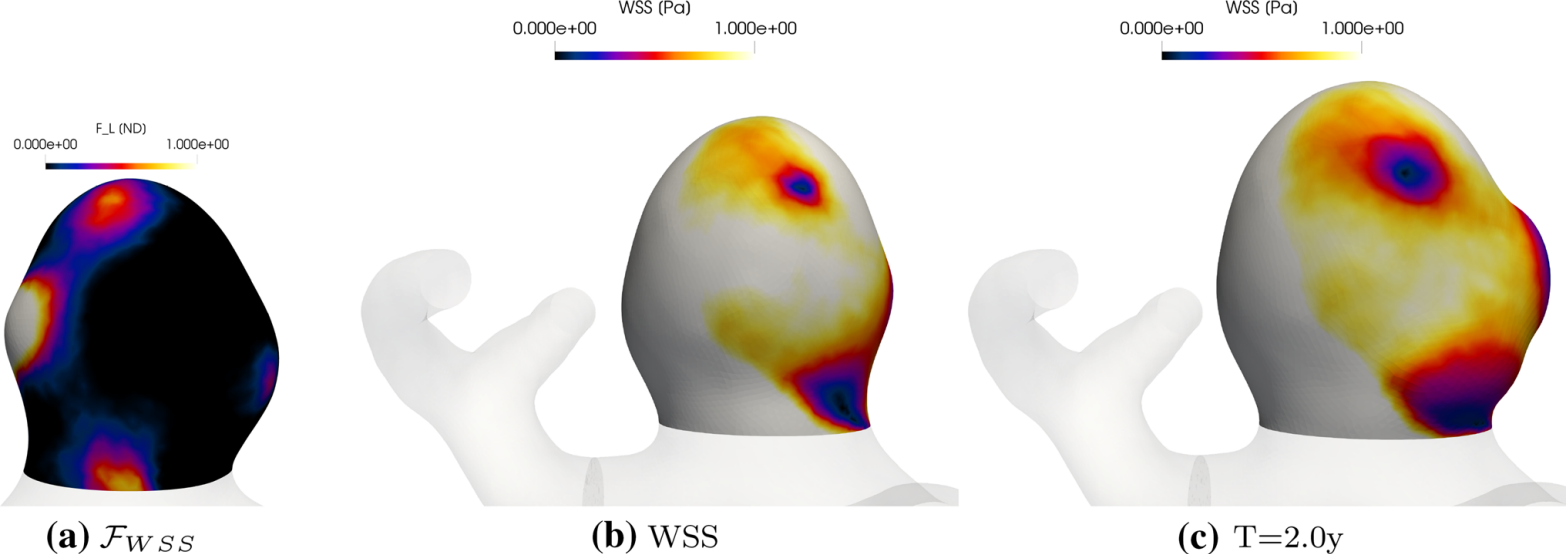
**a** $\mathcal{F}_{\rm{WSS}}$ at $T=0$. WSS at $T=0$ **b** and $T=2$ (c)

Figure 13 illustrates $\mathcal{F}_{\rm{WSS}}$ at $t=0$ and the spatial distributions of WSS at $t=0$ and $t=2$. In regions where the WSS is below 0.5, maximum degradation occurs (indicated by $\mathcal{F}_{\rm{WSS}}$=1). It can be seen that the whole aneurysm sac enlarges in size and a prominant secondary bleb develops on the upstream region of the aneurysm sac where a region of low WSS is located. This is facilitated by a feedback mechanism whereby the regions of low WSS enlarge in size as the aneurysm enlarges (compare Fig. 13b, c).

**Fig. 14:**
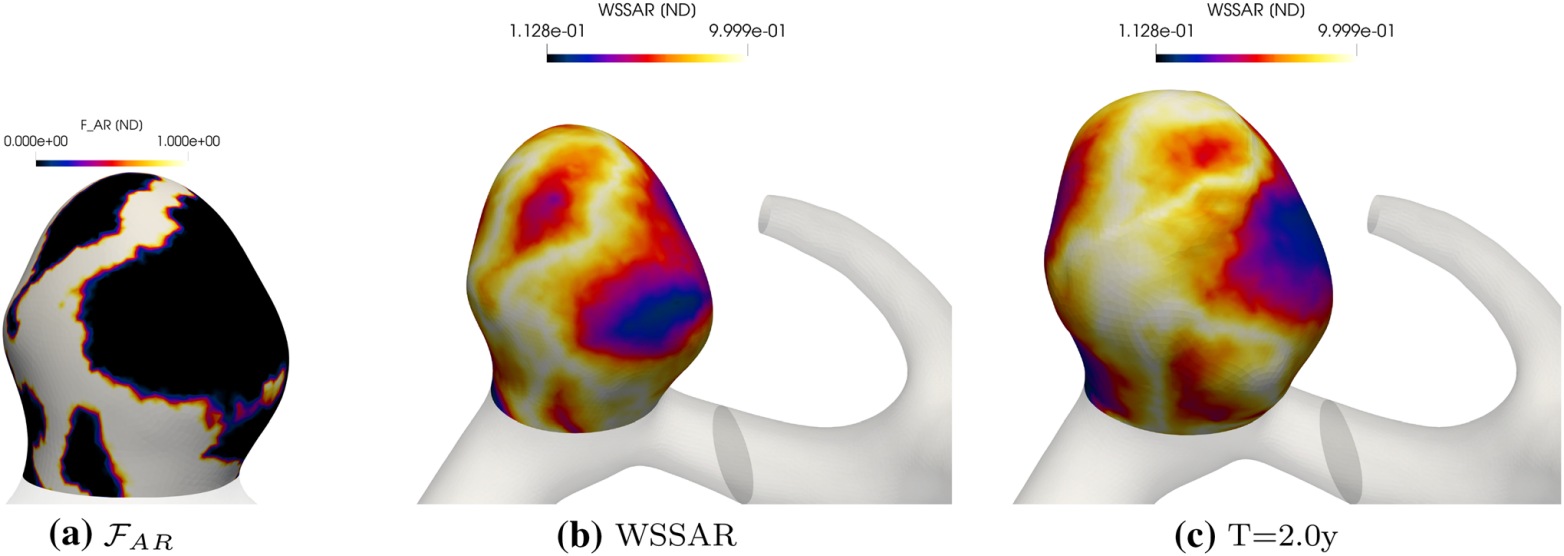
**a** $\mathcal{F}_{\rm{AR}}$ at $T=0$. WSSAR at $T=0$ (**b**) and $T=2$ (**c**)

Figure 14 illustrates $\mathcal{F}_{\rm{AR}}$ at $t=0$ and the spatial distributions of WSSAR at $t=0$ and $t=2$. Regions of high WSSAR are associated with oscillatory flow and we hypothesize that in these regions the endothelium will have an irregular morphology. In these locations, i.e. the white regions of Fig. 14a where $\mathcal{F}_{\rm{AR}}=1$, degradation is maximal. For this example, $\mathcal{F}_{\rm{AR}}$ does not have localized maxima and consequently the whole IA sac is seen to enlarge without the development of focal secondary blebs.

**Fig. 15:**
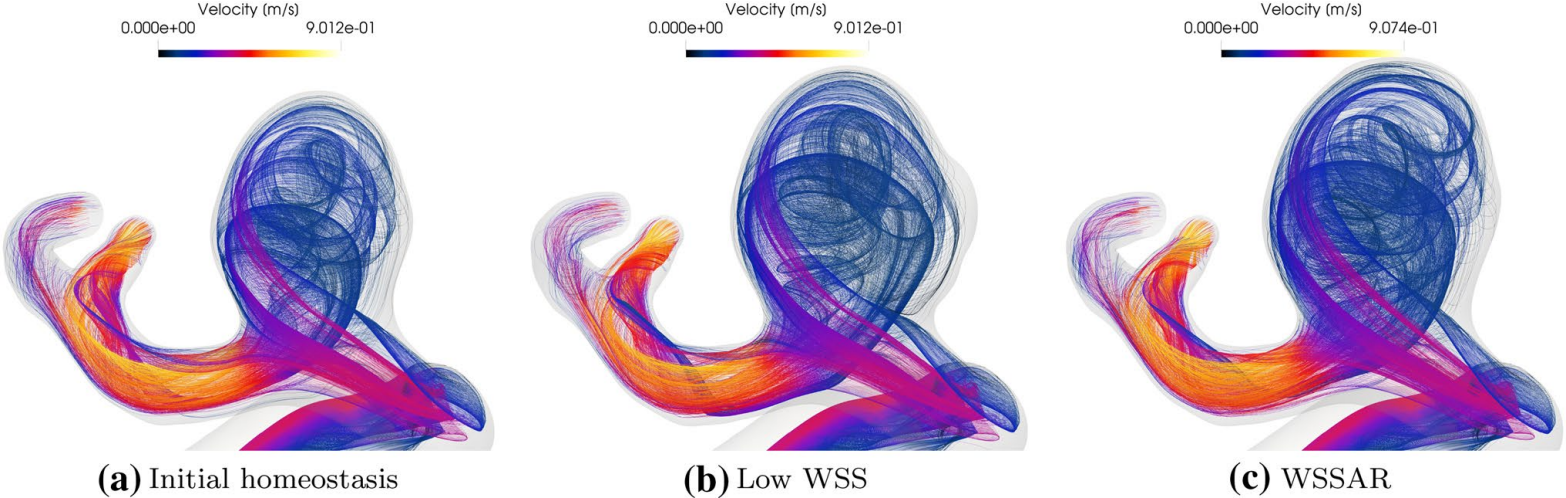
Evolution of streamlines from **a** the initial to the final state for **b** WSS and **c** WSSAR driven enlargement

The impact on the blood flow velocity field of distinct WSS-related degradation hypotheses is evident in Fig. 15, which compares the velocity streamline from the initial homeostasis (common to all degradation methods) to that of each resulting configuration.

### Stabilization

**Fig. 16:**
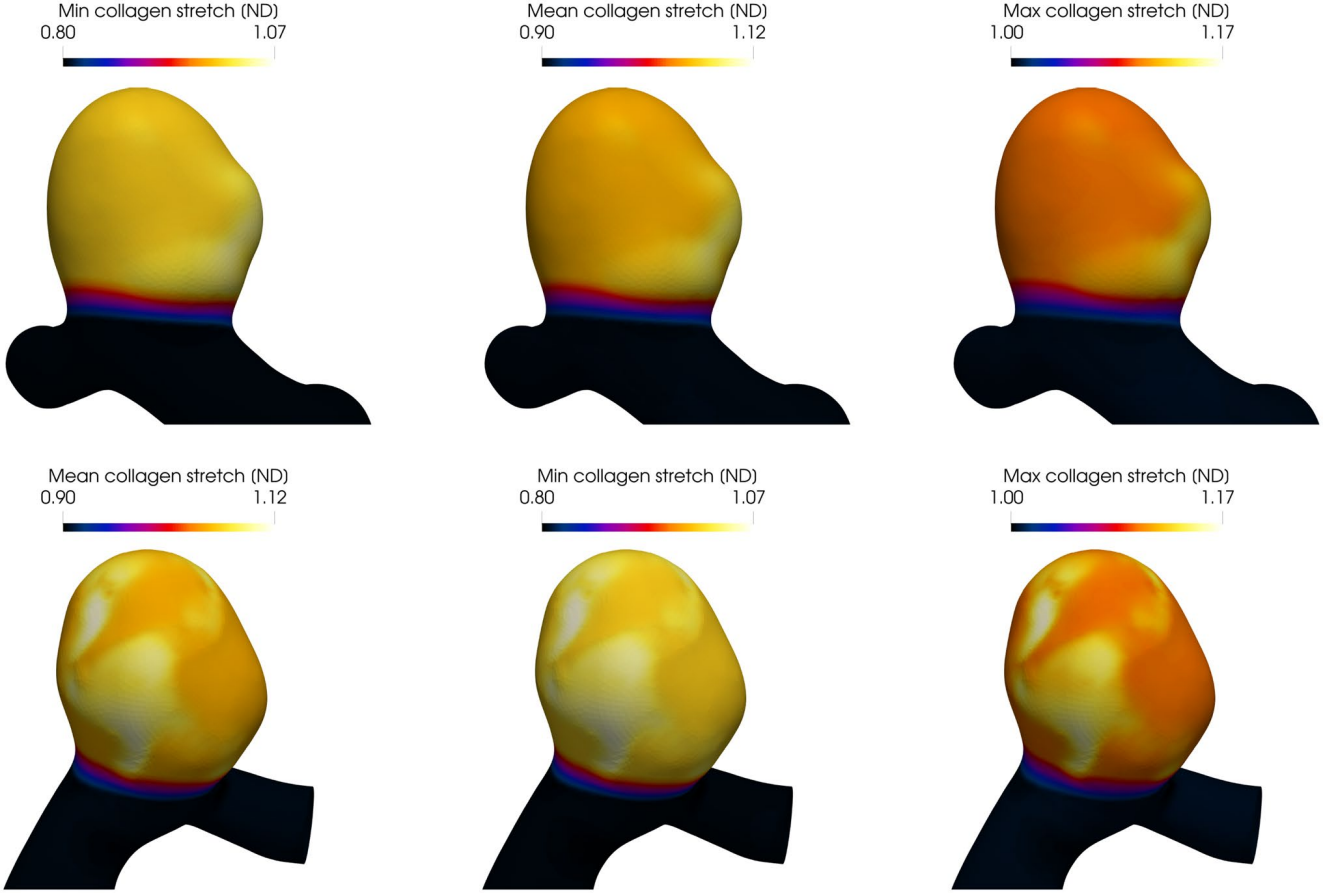
Final homeostatic state of the collagen fiber stretch under the low-WSS hypothesis (first row), and the WSSAR hypothesis (second row). Minimum, mean and maximum collagen stretches are plotted in the first, second, and third column, respectively. Note: in both scenarios, mechanobiological equilibium is achieved via a spatially heterogeneous attachment stretch distribution

Figure 16 illustrates the collagen fiber stretch distributions in the final stabilized state for low-WSS driven enlargement (upper) and high-WSSAR driven enlargement (lower). As material is degraded and the IA sac enlarges, the fiber stretches increase to maintain mechanical equilibrium. As the stabilization mechanism is a point-wise relation, see Eq. 15, the mechanobiological equilibrium at the final homeostatic state contains a spatially-variable attachment stretch field. It can be seen that the magnitudes of the attachment stretch distribution (see Fig.  16) have increased (compare with Fig. 10). More specifically, to stabilize the aneurysm, the attachment stretch distribution evolves from a spatially homogeneous distribution where min/ mode/max attachment stretches of the distribution are 1.01/1.05/1.1 to a spatially heterogeneous distribution where these values increase up to 1.07/1.12/1.17, respectively.

## Discussion

We have presented the first Fluid-Solid-Growth framework to model IA growth and stabilization for personalized (image-based) IA geometries. Two illustrative scenarios to drive IA enlargement are presented: low WSS and complex, oscillatory flow. We propose a novel approach to link endothelial morphology (aligned vs. irregular) to a novel pulsatile flow metric (WSSAR) and subsequently localized degradation of the tissue. Moreover, the model integrates a mechanism to account for aneurysm stabilization, i.e. adaption of the adventitial collagen fabric via remodelling of the collagen fiber attachment stretch distribution.

The model is fully implemented into *Sim4Life*, a state-of-the-art simulation platform for computational life sciences (Neufeld et al. 2013). To our knowledge, *Sim4Life* is the first fully integrative framework for modeling IA evolution of its type: it incorporates user-friendly tools which span from image segmentation to simulation of IA enlargement.

**Fig. 17:**
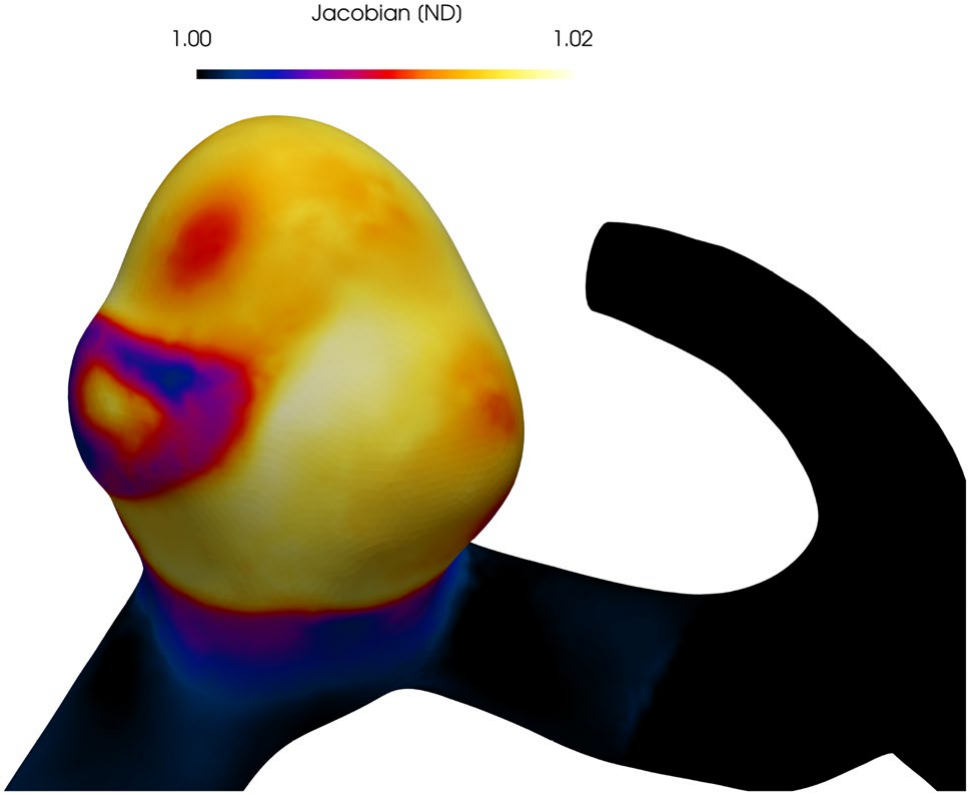
Final pressurized configuration following formation of a secondary bleb. The Jacobian is close to 1 and hence near-incompressibility is achieved

We utilized an isochoric split of the deformation gradient for our finite element (FE) model. It is recognized that this can cause problems for fiber-reinforced materials that lead to FE simulations producing unrealistic behaviour (volumetric swelling) of a material (Sansour 2008; Helfenstein et al. 2010; Gültekin et al. 2019). Essentially, from an energy minimization perspective, at a critical fiber stretch, it becomes energetically more favourable to swell the material as opposed to stretch along the fiber direction (Zdunek et al. 2014). Interestingly, the (isochoric) fiber stretch at which this occurs can be calculated by consideration of the relative stiffnesses of the bulk modulus and embedded fiber (Zdunek et al. 2014). If a material is modeled to have a stiffness that increases exponentially, then—for physiological consistent material parameters—this issue occurs at relatively small deformation, e.g. fiber stretches $< 1.2$. However, if a material has constant stiffness, then a bulk modulus value can be chosen so that the issue does not occur (Zdunek et al. 2014). The constitutive model that we adopt for collagen has a constant stiffness once all fibers are recruited and thus a bulk modulus can be chosen appropriately to maintain incompressibility whilst using an isochoric-split of the deformation gradient; volumetric changes were within 2%, see Fig. 17.

The constitutive model for collagen assumed a linear relationship between the 1st Piola–Kirchoff stress and stretch for individual fibers. A nonlinear relation is applied to model the deformation of the fibers and the total strain-energy of the fiber ensemble requires an integral over the recruitment distribution (Hill et al. 2012). Whilst an exponential strain energy function is often used to capture the gross mechanical response of collagen, the nonlinear behaviour is a consequence of individual fibers being recruited to load over a range of recruitment stresses while the individual fibers have a linear response (see discussion in Zhang et al. (2016). This is found to be suitable for modelling the response of arteries (Bevan et al. 2018). It is of note that the triangular recruitment stretch distribution (and linear fiber response) yields analytic expressions for the total stress of the fiber ensemble,and is therefore straightforward to implement into FE codes.

A typical healthy arterial wall is composed of three layers (intima, media and adventitia—some regions contain internal and external elastic lamina to separate the layers) and several components (ECs, elastin, collagen, proteoglycans, fibrilin, nerves, fibroblast, vasa vasorum, etc.). We assumed that the diseased tissue region features a loss of internal elastin lamina and a degraded extracellular matrix. The constitutive model for reinforced collagenous fiber we assume here is relatively simple to account for the full complexity of the wall constituents and physiological processes which happen in the artery. They could be augmented with models of passive and active SMCs response (Bhogal et al. 2019) (which play an important role in aneurysm formation (Kondo et al. 1998)), angle dispersion of collagen fibers (Holzapfel et al. 2015), and macrophage infiltration (proinflammatory signaling—which are linked to flow and drive aneurysm formation (Harvey et al. 2018) and progression (Frösen et al. 2019), and anisotropic volumetric growth models (Grytsan et al. 2017). In fact, recent experimental research (Cebral et al. 2019) observes that the tissue has a much more complex heterogeneous structure, with regions that have an atherosclerotic nature. More sophisticated models are needed which account for this complex pathophysiology.

The clinical geometry is obtained under physiological loading—however here we considered it to be the unloaded configuration. A pre-stressing algorithm (Weisbecker et al. 2014) is required to compute the actual unloaded configuration, such that the given input geometry is the loaded configuration. Note also that due to the lack of imaging resolution, the wall thickness has to be artificially defined; here we adopted a uniform thickness.

Experiments show that blood exhibits a Newtonian behaviour in most large arteries (Ku 1997), and a non-Newtonian behavior in vessels with a diameter below $100 \mu m$ (Popel and Johnson 2005). Several publications debate the impact of the flow model on numerical modeling because of the particulare nature of blood (Blanco et al. 2006; Xiang et al. 2011; Evju and Mardal 2015; Saqr et al. 2019). The general consensus is that Newtonian and non-Newtonian models are useful, and that their applicability depends on the size of the arterial lumen. Even though (Xiang et al. 2012) reports that Newtonian model can overestimate WSS, (Cebral et al. 2011, 2016) finds no evidence to prefer the non-Newtonian model over the Newtonian one. Because the arteries of the circle of Willis are still *large* with respect to the RBCs size, we followed Steinman and Pereira (2019) in this work and modeled blood as a Newtonian flow, with constant viscosity $\mu$.

ECs morphology is hypothesized to affect wall remodeling (Kaneko et al. 2018). Permeability dysfunction leads to transmigration of leucocytes into the wall and degradation of the extracellular matrix (Babu et al. 2015). Here we proposed a novel approach to predict EC morphology and link it to localized degradation of the wall. For illustration, we implemented this with a novel pulsatile flow metric that quantifies the oscillatory nature of the flow, i.e. WSSAR (Krishna et al. 2020). However other pulsatile flow metrics could be employed for the same purpose, e.g. OSI or high WSS, which were recently correlated to proinflammatory signaling and aneurysm initiation (Frösen et al. 2019). Furthermore, EC morphology is linked to cyclic deformation of the wall (Watton et al. 2011a) and hence the algorithm could be enhanced to include this additional mechanobiological influence. The influence of cyclic deformation on remodelling can be easily included: our framework currently simulate the evolution of systolic and diastolic fields as in Watton et al. (2011a) and already incorporates a monolithic fluid-structure interaction solver.

The mechanisms that lead to IA formation, growth and rupture are incompletely understood. From a CFD perspective, each of these phases appears to be correlated to non-physiological low WSS, high WSS, oscillatory flow and combinations of them (Staarmann et al. 2019). Follow up imaging depicting enlarging IAs obtained from clinical databases (Bijlenga et al. 2019) will assist in generating and testing G&R hypotheses in future applications of the framework.

There is a need to move beyond phenomenological correlations that relate flow metrics to IA stability/growth. More specifically, mechanistic understanding of the governing mechanobiology of IAs is needed to improve management of the disease (Robertson and Watton 2012). We envision that the application of the presented *in silico* framework to *in vitro* and *in vivo* (animal/human) models, e.g. (Kaneko et al. 2018; Cebral et al. 2015, 2016; Wang et al. 2018; Cebral et al. 2018, 2019; Frösen et al. 2019), will be a step towards this goal.

## Summary

This paper presents the first integrative fluid-solid-growth modelling framework with application to modelling enlargement/stabilization of patient-specific IAs. It will provide new mechanistic insight into the mechanobiology of IA disease.
